# Association between intimate partner psychological violence and psychological distress among nurses: The role of personality traits and social support

**DOI:** 10.3389/fpsyg.2022.1038428

**Published:** 2023-01-12

**Authors:** Wentao Huang, Fan Zhang, Xibin Sun, Qing Yu, Jingxin Huang, Yunhui Su, Yutao Lan

**Affiliations:** ^1^Department of Thoracic Surgery, State Key Laboratory of Oncology in South China, Collaborative Innovation Center for Cancer Medicine, Sun Yat-sen University Cancer Center, Guangzhou, China; ^2^Department of Nursing, State Key Laboratory of Oncology in South China, Collaborative Innovation Center for Cancer Medicine, Sun Yat-sen University Cancer Center, Guangzhou, China; ^3^School of Nursing, Guangdong Pharmaceutical University, Guangzhou, China; ^4^Department of Emergency Medicine, The First Affiliated Hospital of Guangdong Pharmaceutical University, Guangzhou, China; ^5^School of Public Health, Guangdong Pharmaceutical University, Guangzhou, China

**Keywords:** intimate partner violence, nurse, psychological violence, personality traits, social support, psychological distress

## Abstract

**Aim:**

This study proposes investigating the risk and protective factors of intimate partner (IP) psychological violence and psychological distress to better promote psychological wellbeing for nurses and health outcomes for patients.

**Design:**

A cross-sectional study.

**Method:**

This cross-sectional study was carried out chiefly in Guangdong, Hunan, and Shaanxi provinces, in the east, central, and west of the Chinese economic areas, respectively. It was conducted in October 2021 using convenience sampling. A total of 843 nurses were eligible for the final analysis. Single-factor linear regression models were used to identify potential factors associated with IP psychological violence and psychological distress. In addition, the structural equation model was used to explore the role of personality traits and social support in the association between IP psychological violence and psychological distress.

**Results:**

The predictors for the score of IP psychological violence among nurses were participants' married status, contact frequency with a partner, perceived past-year psychological and physical violence experience, the alcohol consumption of partners, and personality traits and social support of partners. Moreover, the alcohol consumption of participants, the past-year experience of IP psychological violence, the score of psychological violence, personality traits, social support, and the personality traits of partners were associated factors affecting the psychological distress of nurses. In the structural model, the personality trait of partners had a direct pathway to psychological violence and social support. The results demonstrated that psychological violence significantly increased psychological distress.

**Conclusion:**

Personality traits and social support are essential factors influencing the relationship between IP psychological violence and psychological distress.

**Impact:**

The findings of this study emphasize the possibility and importance of identification and intervention for reducing IP psychological violence based on personality traits and social support.

## 1. Introduction

Nurses are an essential part of the healthcare system and strongly contribute to better patient outcomes. Therefore, factors that affect the quality of care from nurses should be studied, including professional competence and psychological state. However, a high prevalence of adverse psychological conditions among staff nurses has been reported in the past years. Studies revealed that more than 92.3% of nurses (Feng et al., 2018) reported the presence of psychological distress and 38.5% (Huang et al., 2021) had moderate-severe psychological distress. These results may be attributed to multiple stressors, including work overload, nurse-patient, and work-family conflicts (Hao et al., 2015; López-López et al., 2019). Such conflicts and violence from an intimate partner or family can significantly affect the psychological wellbeing of nurses. Those conflicts have been demonstrated to increase depressive symptoms and burnout among nurses by destroying the psychological capital of survivors (Hao et al., 2015), seriously affecting their physical and mental health, and further reducing their efficiency at work (MacGregor et al., 2022). Trauma from intimate partner violence (IPV) may compound the challenge for patient healthcare, thereby reducing the quality of care and resulting in higher medical risks. Nurses serve as care and intervention providers for the victims who suffer from IPV. However, there is evidence that health professionals are also experiencing a high prevalence of IPV (Carmona-Torres et al., 2017, 2018, 2019; McLindon et al., 2018). Health professionals, especially nurses, are more vulnerable to IPV and have barriers to seeking support compared with the general population (Dheensa et al., 2022). However, nurse experiences of IPV have been ignored because the subjects in most studies are pregnant women or LGBT people (Chisholm et al., 2017; Lutgendorf, 2019; Swan et al., 2021) rather than nurses or face violence in the workplace (Liu et al., 2019). Therefore, considering the above, it is important to determine the prevalence of IPV among nurses, and having a better understanding of the risk and protective factors of IPV could promote better psychological wellbeing for nurses and health outcomes for patients.

## 2. Background

### 2.1. Types, prevalence, and consequences of IPV

Intimate partner violence is referred to as behavior and includes various forms of physical, sexual, and psychological violence by a current or former intimate partner (Garcia-Moreno et al., 2006; Chisholm et al., 2017). It constitutes a major global problem as many people, especially women, are affected throughout the world, and it was estimated that 27% (uncertainty interval: 23–31%) and 13% (10–16%) of women aged 15–49 years have experienced lifetime and past-year IPV, respectively (Sardinha et al., 2022). Although the prevalence of IPV is relatively low in East Asia [19% (11–32%) and 8% (3–17%) for lifetime and past-year IPV] (Sardinha et al., 2022), a meta-analysis found a high prevalence of IPV [7.7% (95% CI: 5.6–10.1%)] among pregnant women in China, which was the highest reported number in Asia (Wang et al., 2017). IPV may contribute to a series of physical and mental traumas and sequelae (Sugg, 2015), constituting an essential determinant of the mental health of an individual. Moreover, it is well-established that IPV is associated with a higher incidence of anxiety, depression, psychological distress, suicide attempts, and severe mental illness (Devries et al., 2013; Sugg, 2015; Chandan et al., 2020). The association between IPV and adverse psychological wellbeing is bidirectional (Shen and Kusunoki, 2019). Thus, women who reported psychological distress are at a higher risk of suffering psychological violence (Oram et al., 2019; Keynejad et al., 2020). However, the prevalence of psychological violence, also known as cold violence, is increasing, which is even higher than physical violence and sexual violence (Dokkedahl et al., 2019; Gibbs et al., 2020; Chen and Chan, 2021; Occean et al., 2021). A study conducted in rural areas of China reported that 26.58, 7.66, and 3.20% of women have experienced psychological, physical, and sexual violence from an intimate partner (Hou et al., 2018). Psychological violence can be perpetrated in various forms, including threats, verbal abuse, accusations, and/or manipulation. Although it has a high prevalence, harm from psychological violence is often invisible and neglected (Arriaga and Schkeryantz, 2015). Compared to physical violence and sexual violence, victims of psychological violence are at a higher risk of depression (Tiwari et al., 2008). Additionally, psychological violence can have long-term effects on the self-esteem and identity of women, making the injury linger and hard to recover from Matheson et al. (2015).

### 2.2. Individual, demographic, and social factors associated with intimate partner violence

Intimate partner violence is a complex crisis attributed to multiple risk factors, including sociodemographic characteristics, relationship quality, religion, and social support. The social-ecological theory reveals the complex relationship between individual risk factors (including personal and partner factors), family, community, societal level, IPV, and mental health (Tekkas Kerman and Betrus, 2020). Thus, this complexity emphasizes the interaction between the individual and society on health outcomes (Campbell et al., 2009; Mojahed et al., 2020; Tekkas Kerman and Betrus, 2020). Personality traits are well-established to be associated with aggressive behavior and may enhance or inhibit aggressive moods and actions (Bettencourt et al., 2006; Barlett and Anderson, 2012). Openness, agreeableness, and neuroticism are related to bold emotions and physical aggression^30^. Conscientiousness was significantly and positively correlated with the gray matter volume in the right inferior frontal gyrus, which has revealed the relationship between personality traits and emotion regulation (Chen et al., 2018). Therefore, the personality traits of a partner might likely help to predict individuals with a higher risk of IPV. Personality traits were also strongly associated with the mental health of individuals (Nouri et al., 2019; Huang et al., 2021). Individuals characterized by high neuroticism, low extraversion, and low conscientiousness are associated with a greater risk of depression (Nouri et al., 2019). Similarly, the situation has also been described among physicians (Gramstad et al., 2013) and nurses (Warbah et al., 2007). Exploring the personality traits associated with the exposure and enforcement of IPV may help identify individuals who may be at risk for IPV and subsequent mental health problems.

The demographic factors of individuals were related to the prevalence of IPV, namely, gender, education level, employment status, personalities of individuals and partners, and alcohol consumption (Jewkes, 2002; Hou et al., 2018). Although women are regarded as victims in most situations, evidence showed gender symmetry regarding minor physical violence and psychological violence (Hou et al., 2018). Some studies pointed out that education level is a crucial predictor of IPV, and a high education level seems to protect women from suffering IPV and IP psychological violence (Debono et al., 2017; Cau, 2020; Choi et al., 2021). However, another study indicated that the education level of female victims could not predict or protect against severe IPV and homicide (Carmichael et al., 2019). The link between alcohol use/abuse and partner violence is well-established (Jewkes, 2002). Alcohol consumption is always linked to violence (Miczek et al., 2015). Therefore, men who frequently consume alcohol are more likely to commit all forms of IPV against their IP. Furthermore, alcohol consumption was significantly higher before IPV and had symmetry between men and women (Murphy et al., 2005; Foran and O'Leary, 2008; Kaufmann et al., 2014). Consequently, sociodemographic and lifestyle factors have been demonstrated as critical predictors of IPV.

The occurrence of IPV can be explained by the complex interplay between individual, relationship, community, and societal factors (Tekkas Kerman and Betrus, 2020). Social support is an essential factor in this interaction. Inadequate social support from the family and society is considered a risk factor affecting IPV and depressive symptoms (Fu et al., 2021), and high social support can be protective against some of the negative impacts of IPV and mental health (Escribà-Agüir et al., 2010; Abbas et al., 2019; Choi et al., 2021; Fedina et al., 2021). Individuals with weakened neighborhood relationships were associated with higher IPV levels and were exposed to a greater risk of psychological distress and suicide risk (Fedina et al., 2021). In addition, women exposed to IPV but with an extensive family network have a 29% lesser chance of poor self-perceived health status than those in a small family network (Escribà-Agüir et al., 2010). Social support was found to mediate between marital cohesion and depression (Abbas et al., 2019), which indicated that social support might mitigate the impact of conflict in intimate relationships. Thus, establishing strategies to improve social support may reduce IPV and subsequent adverse outcomes.

However, the prevalence of IP psychological violence and predictive and protective factors on IP psychological violence and psychological distress among nurses remain unknown. Therefore, it is unclear whether the risk factors of mutual violence are similar to those of IPV perpetration or victimization.

## 3. Study

### 3.1. Objective

This study explores the determinants associated with IP psychological violence and psychological distress among nurses. It examines the role of personality traits and social support in the relationship between IP psychological violence and psychological distress. Furthermore, this study explores whether social support can reduce the risk of IP psychological violence and psychological distress after suffering IP psychological violence.

### 3.2. Design

This is a cross-sectional study with a descriptive and correlational design using self-reported questionnaires and data instruments.

## 4. Methods

### 4.1. Data collection

This cross-sectional study was carried out chiefly in the Guangdong, Hunan, and Shaanxi provinces, in the east, central, and west of the Chinese economic areas. Eligible nurses and nursing supervisors were recruited to complete an online survey, using the Questionnaire Star platform (Changsha Ranxing Information Technology Co. Ltd.) through WeChat software (an instant messaging software widespread use in China) or e-mails. All electronic questionnaires were sent *via* a private letter from the research team or the supervisor of the subject. The questionnaires were issued to all potential subjects but were not artificially selected or mandatory. Participants have been informed that the study is not being conducted by their supervisors and institutions; the data will only be used for academic research and will not affect their work. Participants were required to answer all the questions in the questionary before the submission, and no personal identification information was collected.

### 4.2. Participants

The potential eligible participants included in this study have the following characteristics: (1) age ≥ 18 years; (2) a registered nurse; (3) have or used to have at least one intimate partner; and (4) voluntarily consented to the survey. A total of 1,616 participants received the questionnaires initially, and 843 participants were eligible for final analysis after excluding 773 people who reported no intimate partner. The potential number of variables for this study was 39. As a result, the sample size was higher than 20 subjects per variable, which was adequate for conducting a multivariate linear regression analysis based on the statistical method study (Austin and Steyerberg, 2015).

### 4.3. Ethical consideration

Ethical approval [No. (2021)-165] was granted by the institutional review board of the First Affiliated Hospital of Guangdong Pharmaceutical University in China. All participants have been informed of the (1) research objectives and procedures; (2) potential benefits, cost, and risk; and (3) the privacy policy before they answer the questionnaire. The participants could withdraw from the survey at any time, and data were erased in this case. Completing and submitting the electronic questionnaire was considered informed consent, indicating voluntary participation in the study.

### 4.4. Validity and reliability

All the instruments used in this study were based on previous studies, had validated scales, and were associated with high validity. Reliability and validity tests were used to confirm validation for each scale in this study.

### 4.5. Measures

#### 4.5.1. Demographic characteristics

A self-developed questionnaire was used to collect demographic variables, such as age, sex, children situation (have or not), marital status (married, unmarried, cohabit, or non-cohabit), educational levels (technical secondary school, junior college, undergraduate, or postgraduate), alcohol consumption status of participants and partners (lifetime abstainer, former drinker, < 1 time per week, and ≥1 time per week), frequency of contact (always, often, occasionally, and hardly ever), and self-reported 1-year violence experience (psychological violence, physical violence, and sexual violence).

#### 4.5.2. Personality traits

The Chinese version of the ten-item personality inventory questionnaire (TIPI-C) was used (Li, 2013). The TIPI is a brief personality assessment scale that could quickly assess the big-five personality traits dimensions [i.e., extraversion, agreeableness, conscientiousness, emotional stability (antithesis of neuroticism), and openness], scored on a 7-point Likert-type scale, with one as “*disagree strongly”* and seven as “*agree strongly*.” Each dimension consists of two reverse items; one item represents a positive trait (e.g., extraverted and enthusiastic) and the other a negative trait (e.g., reserved and quiet). The scores for every five dimensions were summed up with two items; higher scores for each dimension, indicating each positive trait, are preferred. The Cronbach's alpha coefficient for TIPI-C used in this study was 0.733. We asked the participants to rate their scores on a big-five personality trait questionnaire for both themselves and their partners. Therefore, in this study, the personality traits of the partners were reported by the participants and not measured directly by their partners.

#### 4.5.3. Social support

Social support was measured using the social support rating scale (SSRS) developed by Xiao (1994), which is widely used in the Chinese population. The SSRS comprises three subscales, namely, subjective support (S-SS), objective support (O-SS), and utilization of social support (U-SS). A higher score indicates that a participant perceived or received more support from family, friends, or organizations and how actively the individual utilized that support, respectively. The Cronbach's α for SSRS used in this study was 0.794.

#### 4.5.4. Intimate partner psychological violence

Intimate partner psychological violence was assessed using the Chinese domestic cold violence scale, a seven-item instrument developed by Yan et al. (2019), which can measure the severity of psychological violence by the spouse or intimate partner. Participants were asked to report how frequently the events related to psychological violence happened (e.g., insulting, isolating, distant, and indifferent), using a 4-point scale (from 1 for never happened to four for always happened). Items were summed, and a higher score indicated more severe experiences of psychological violence. The Cronbach's alpha for the scale was considered acceptable (0.912) in the present study. To validate the effectiveness of the scale for screening IPV, the ROC-curve analysis was computed to test the classification accuracy of participants with self-reported past-year psychological, physical, and sexual violence from an intimate partner, and the results indicated a high accuracy (AUC = 0.824, 0.786, and 0.787, respectively), which showed that the scores of intimate psychological violence measured by the scale were significantly and highly correlated with self-reported past-year IPV occurrence of the participants.

#### 4.5.5. Psychological distress

The Kessler Measure of Psychological Distress (K10) was used to measure psychological distress (Kessler et al., 2003). Participants were asked about the frequency of experiencing symptoms in the past month, such as tiredness, nervousness, and depression. Each item was rated on a 5-point Likert scale (from 1 for none of the time to five for all the time), with a higher score indicating greater severity of psychological distress. The Cronbach's α obtained in this study was 0.953.

### 4.6. Data analyses

Descriptive statistics were used to report the distribution of the characteristics of the participants. Single-factor linear regression models were used to identify potential factors associated with psychological violence and psychological distress. A *p* < 0.05 in the adjusted model was imported into the final model. Finally, a structural equation model (SEM) with a bootstrap estimate based on 1,000 bootstrap samples was used to explore the role of personality traits and social support in the association between psychological violence and psychological distress. The model fit was evaluated using fit indices of mean square error of approximation (RMSEA), comparative fit index (CFI), Tucker-Lewis index (TLI), and standardized root mean square residual (SRMR). Descriptive statistics analysis and linear regression modeling were performed using STATA version 15.0 (Stata Corp., College Station, TX, USA). In contrast, SEM analysis was performed using Amos 26.0 (Amos Development Corporation, Meadville, PA, USA). The significant level was set to α = 0.05 (two-tailed).

## 5. Results

### 5.1. Demographic characteristics of participants

Table 1 displays the demographic characteristics of participants in this study. Of the 843 nurses (mean age = 34.35 years) who were enrolled in the final analysis, 809 (96.0%) were women, 698 (82.8%) were married and cohabiting with a partner, and 659 (78.2%) were undergraduates. A total of 234 (27.8%) participants and 507 (60.1%) of their partners reported having alcohol more than one drink per week. Of the participants, 191 (22.7%) reported suffering psychological violence from their intimate partner in the past year. In addition, there were 28 (3.3%) and 10 (1.2%) participants who also experienced physical violence and sexual violence, respectively. The correlation analysis results showed that social support, psychological violence, and psychological distress were significantly associated with each other (*p* < 0.001, Table 2).

**Table 1:** Demographic characteristics, *n* = 843.

**Characteristics**	**Category**	***n*/X**	**%/SD**
Age, Mean ± SD		34.35	7.55
Women, *n* (%)		809	96.0
Have children, *n* (%)		662	78.5
Married status, *n* (%)	Marriage-cohabit	698	82.8
	Married, non-cohabit	32	3.8
	Unmarried, cohabit	28	3.3
	Unmarried, non-cohabit	85	10.1
Educational level, *n* (%)	Technical secondary school	14	1.7
	Junior college	146	17.3
	Undergraduate	659	78.2
	Postgraduate	24	2.9
Alcohol consumption, *n* (%)	Lifetime abstainer	588	69.8
	Former drinker	21	2.5
	< 1 day/week	218	25.9
	≥1 day/week	16	1.9
Partner alcohol consumption, *n* (%)	Lifetime abstainer	308	36.5
	Former drinker	28	3.3
	< 1 day/week	384	45.6
	≥1 day/week	123	14.6
Contact frequency, *n* (%)	Always	444	52.7
	Often	277	32.9
	Occasionally	75	8.9
	Hardly ever	47	5.6
1 year violence experience, *n* (%)	Psychological violence	191	22.7
	Physical violence	28	3.3
	Sexual violence	10	1.2

**Table 2:** Descriptive and bivariate correlation analysis for social support, psychological violence, and psychological distress.

	**Mean ±SD**	**1**	**2**	**3**	**4**	**5**	**6**
Subjective support	24.40 ± 4.62	–	0.428^***^	0.455^***^	0.916^***^	0.314^***^	0.391^***^
Objective support	8.74 ± 2.16		–	0.308^***^	0.678^***^	0.224^***^	0.260^***^
Usage of support	8.01 ± 2.01			–	0.677^***^	0.203^***^	0.312^***^
Social support (total)	41.15 ± 7.05				–	−0.332^***^	0.425^***^
Psychological violence	9.36 ± 3.75					–	0.373^***^
Psychological distress	9.89 ± 8.44						–

^***^ *p* < 0.001.

### 5.2. Factors affecting psychological violence

Participants who were married but not cohabiting as a couple (regression coefficients β = 1.08, 95 %CI 0.04–2.12) and contacted hardly (β = 2.88, 95% CI 1.99–3.78) reported higher IP psychological violence experiences compared with those who got married and cohabited and contacted frequently. In addition, participants whose intimate partners had more than one drink per week were at a higher risk of psychological violence (β = 0.69, 95% CI 0.10–1.29) than a lifetime abstainer. Additionally, those having experience of past-year psychological (β = 2.74, 95% CI 2.23–3.25) and physical violence (β = 2.67, 95% CI 1.56–3.78) reported significantly higher psychological violence experience. However, agreeableness, emotional stability for an intimate partner, objective support, and subjective support from social support may be protective factors against psychological violence (Table 3, Supplementary Table 1).

**Table 3:** Linear regression on selected associated factors for psychological violence among Chinese nurses.

**Factors**	**Unadjusted model**	**Fully adjusted model**	**Final adjusted model**
	**β (95% CI)**	**β (95% CI)**	**β (95% CI)**
Age (continuous)	0.06 (0.03, 0.09)	0.05 (0.02, 0.08)	0.06 (0.03, 0.08)
**Married status (ref: marriage-cohabit)**			
Married, non-cohabit	4.42 (3.13, 5.72)	1.19 (0.13, 2.25)	1.08 (0.04, 2.12)
**Partner's alcohol consumption (ref: lifetime abstainer)**			
≥1 day/week	1.78 (1.00, 2.55)	0.65 (0.00, 1.29)	0.69 (0.10, 1.29)
**Contact frequency (ref: always)**			
Often	1.41 (0.89, 1.93)	0.69 (0.25, 1.13)	0.79 (0.36, 1.22)
Hardly ever	5.78 (4.73, 6.82)	2.66 (1.75, 3.57)	2.88 (1.99, 3.78)
**Past year violence experience (ref: no)**			
Psychological violence	4.51 (3.98, 5.03)	2.68 (2.16, 3.20)	2.74 (2.23, 3.25)
Physical violence	6.39 (5.04, 7.74)	2.18 (0.99, 3.37)	2.67 (1.56, 3.78)
**Partner's personality traits (continuous)**			
Agreeableness	−0.70 (−0.79, −0.60)	−0.20 (−0.32, −0.07)	−0.21 (−0.32, −0.09)
Emotional stability	−0.68 (−0.78, −0.58)	−0.16 (−0.28, −0.04)	−0.18 (−0.30, −0.07)
**Social support (continuous)**			
Objective support	−0.39 (−0.50, −0.27)	−0.16 (−0.27, −0.06)	−0.16 (−0.26, −0.06)
Subjective support	−0.25 (−0.31, −0.20)	−0.09 (−0.15, −0.04)	−0.09 (−0.14, −0.04)
**Model Fit**			
Adjusted *R^2^* (%)		44.59	44.72
*F*		20.93	43.58
*p*		< 0.001	< 0.001

Variables not included in the final model are omitted, with full results in Supplementary Table 1.

Ref, reference variable in the regression model.

Fully adjusted model: adjusted for age, sex, having children, married status, educational level, alcohol consumption, alcohol consumption of partner, contact frequency, past-year violence experience (psychological violence, physical violence, and sexual violence), personality traits of participants, personality traits of partners, social support.

The final adjusted model eliminated the variables that were not statistically significant in the fully adjusted model.

### 5.3. Factors affecting psychological distress

Former drinkers (β = 3.90, 95% CI 0.86–6.94) and those who drink alcohol more than 1 day a week (β = 4.91, 95% CI 1.45–8.38) were experiencing higher psychological distress than lifetime abstainers. In addition, participants with a higher score on IP psychological violence were in more significant psychological distress (β = 0.41, 95% CI 0.25–0.56). The emotional stability of participants, subjective support from social support, and conscientiousness of intimate partners may be protective factors for psychological distress (β < 0, *p* < 0.05; Table 4, Supplementary Table 2).

**Table 4:** Linear regression on selected associated factors for psychological distress among nurses.

**Factors**	**Unadjusted model**	**Fully adjusted model**	**Final adjusted model**
	**β (95% CI)**	**β (95% CI)**	**β (95% CI)**
Age (continuous)	−0.09 (−0.17, −0.02)	−0.05 (−0.12, 0.03)	−0.03 (−0.10, 0.03)
**Alcohol consumption (ref: lifetime abstainer)**			
Former drinker	5.60 (1.95, 9.25)	4.27 (1.17, 7.37)	3.90 (0.86, 6.94)
≥1 day/week	5.90 (1.73, 10.06)	4.72 (1.09, 8.34)	4.91 (1.45, 8.38)
**One year violence experience (ref: no)**			
Psychological violence	6.05 (4.75, 7.35)	1.29 (−0.08, 2.65)	1.23 (−0.09, 2.55)
Psychological violence score (continuous)	0.84 (0.70, 0.98)	0.38 (0.21, 0.55)	0.41 (0.25, 0.56)
**Participant's personality traits (continuous)**			
Emotional stability	−1.63 (−1.85, −1.41)	−0.76 (−1.04, −0.49)	−1.02 (−1.25, −0.79)
**Partner's personality traits (continuous)**			
Conscientiousness	−1.19 (−1.41, −0.97)	−0.27 (−0.52, −0.01)	−0.36 (−0.58, −0.13)
**Social support (continuous)**			
Subjective support	0.06 (−0.83, −0.6)	−0.24 (−0.38, −0.11)	−0.32 (−0.44, −0.20)
**Model fit**			
Adjusted *R^2^* (%)		33.23	32.40
*F*		12.97	45.83
*p*		< 0.001	< 0.001

Variables not included in the final model are omitted, with full results in Supplementary Table 2.

Ref, reference variable in the regression model.

The fully adjusted model adjusted for age, sex, having children, married status, educational level, alcohol consumption, alcohol consumption of partners, contact frequency, past-year violence experience (psychological violence, physical violence, and sexual violence), psychological violence score, personality traits of the participant, personality traits of partner, social support.

The final adjusted model eliminated the variables that were not statistically significant in the fully adjusted model.

### 5.4. Structural equation model for the association between variables of interest

**Figure 1:**
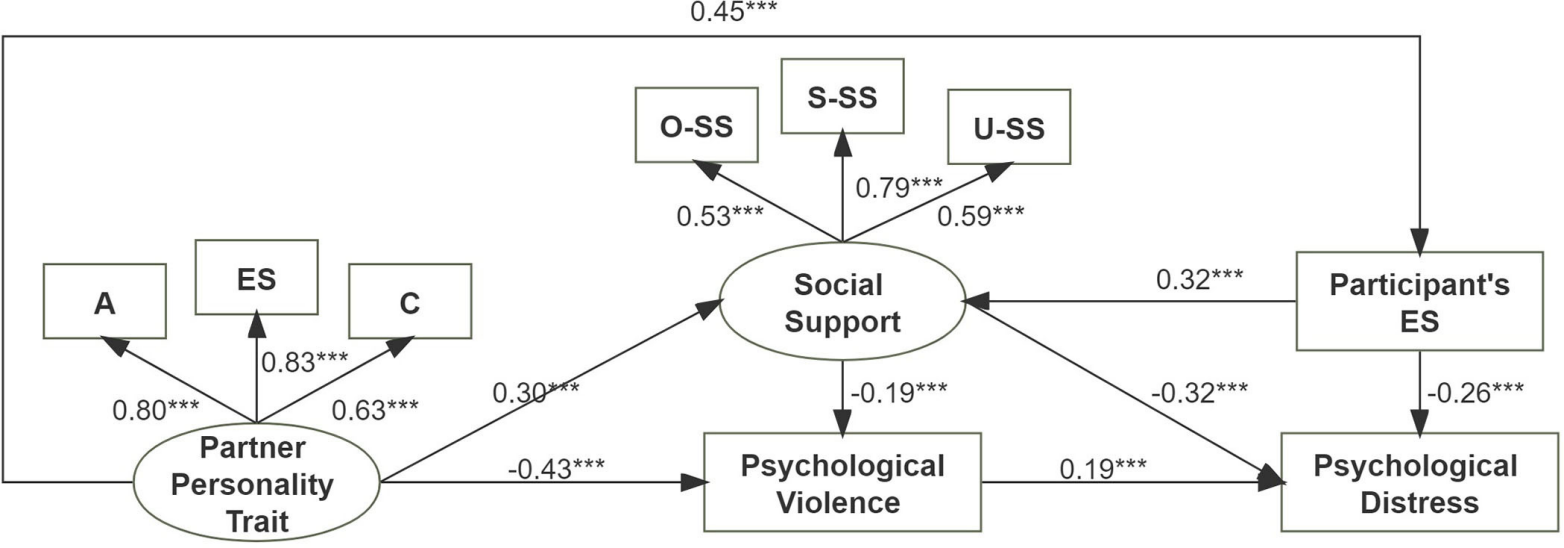
Structural equation model for the relationship between personality traits, social support, psychological violence, and psychological distress. A, agreeableness; ES, emotional stability; C, conscientiousness; S-SS, subjective support; O-SS, objective support; U-SS, utilization of social support. Values are standardized regression coefficients. ***p < 0.01.

According to the abovementioned results, SEM was conducted to determine the relationship between personality traits, social support, psychological violence, and psychological distress. The obtained results provided full support for our hypothesis. Precisely, personality traits from an intimate partner may predict the occurrence and severity of psychological violence (standardized regression coefficient β′ = −0.43, *p* < 0.01). Nurses who have a highly agreeable, emotionally stable, and conscientious intimate partner are less likely to suffer psychological violence and therefore receive more social support (β′ = 0.30, *p* < 0.01). In addition, social support may be enhanced for emotionally stable participants. Thus, greater social support directly evidenced a negative association with psychological distress (β′ = −0.32, *p* < 0.01) and indirectly through reductions in psychological violence (Figure 1, Supplementary Table 3).

## 6. Discussion

This cross-sectional study yielded several significant findings and revealed that the marital status of participants, contact frequency with a partner, perceived past-year psychological and physical violence experience, alcohol consumption of a partner, personality traits of a partner, and social support were predictors for the score of IP psychological violence among nurses. Moreover, the alcohol consumption of participants, the past-year experience of IP psychological violence, the score of psychological violence, personality traits, social support, and personality traits of a partner were associated factors affecting the psychological distress of nurses. In the structural model, the personality trait of a partner is directly associated with psychological violence experience and social support of participants. Psychological violence has significantly increased psychological distress. Therefore, the emotional stability of participants may be advantageous for increasing social support and reducing psychological distress. Additionally, social support affects psychological distress directly and indirectly by decreasing psychological violence. Regarding the prevalence of IP psychological violence among Chinese nurses, some studies focus on nurses and aim to reduce the risk of IP psychological violence and its mental health consequence.

The relationship between the big-five personality traits and IPV among nurses is less studied. This study provides further evidence that the personality traits of partners, especially emotional stability followed by agreeableness, may predict the occurrence of psychological violence. Partners with higher agreeableness, conscientiousness, and emotional stability were less likely to commit psychological violence and were associated with higher social support for participants. However, no association was found between extraversion, openness, and IP psychological violence. These findings were consistent with a previous study that suggested that agreeableness, conscientiousness, and emotional stability of a partner were associated with less conflict in married couples (Buss, 1991) and higher marital satisfaction (Gattis et al., 2004). The present study also partially agrees with a study that suggests that agreeableness, extraversion, and neuroticism may predict IPV severity (Yalch et al., 2020). Another nationally representative study on the adolescent population indicated that openness, extraversion, and neuroticism were risk factors associated with IPV (Ulloa et al., 2016). These inconsistencies may be attributed to the only IP psychological violence investigated in the present study. Regardless of the inconsistencies, neuroticism (emotional stability) was found to be a strong predictor of committing IPV in most studies (Buss, 1991; Ulloa et al., 2016; Yalch et al., 2020). Individuals who are more neurotic and emotionally unstable may have more aggressive emotions, be unable to control their emotions, and consequently adopt more verbal abuse, psychological violence, as well as physical violence to their IP. Low agreeableness was associated with violence in both genders and was mediated by alcohol consumption in men (Jones et al., 2020). Therefore, reducing alcohol consumption in men would significantly reduce IP psychological violence. Conscientiousness correlates with emotion regulation in a neuroanatomical basis study (Chen et al., 2018). Thus, the results showed that some personality traits are unique predictors of IP psychological violence and may help identify high-risk populations with IP psychological violence.

The current study also revealed that social support might reduce psychological distress by decreasing IP psychological violence. Social support is a key ecological determinant for the interaction of life events and mental health outcomes (Campbell et al., 2009; Tekkas Kerman and Betrus, 2020). This study demonstrated that social support from neighborhood and family members could predict depressive symptoms and hopelessness among women who experienced IPV (Pickover et al., 2021). Social support from family members, friends, and neighbors also helps to reduce the incidence of IPV and perinatal depressive and anxiety symptoms after suffering from IPV (Navarrete et al., 2021). Additionally, social support may be conducive to the improvement of post-traumatic growth and self-identification processes, a prompt for an individual seeking favorable personal resolutions positively after experiencing trauma from IPV (Žukauskiene et al., 2021). However, it is a possible kind of social isolation that women in abusive relationships may encounter if they were in a small social network and obtained less support against IPV from the social relationship (Katerndahl et al., 2013). Therefore, an interventional strategy that helps individuals rebuild and expand social networks would be significant.

### 6.1. Implications and limitations

The study provides evidence of the high prevalence of IP psychological violence in Chinese nurses and identifies multiple risk factors affecting IP psychological violence at the individual, interactional, and social levels. Nurses who experienced IP psychological violence reported a higher level of psychological distress. Considering the potential risk of IP psychological violence for nurses, this study suggests that a program that identifies IP psychological violence and provides social support interventions specific to the nurse population is necessary to promote better mental health. These strategies can potentially benefit nurses who are experiencing IP psychological violence by establishing available and clear guidelines for referrals and counseling policies that are timely and effective.

However, the present study has several limitations that should be mentioned. First, the current study sample was recruited *via* a network using an electronic questionnaire. Individuals who experience IPV may avoid answering the questionnaire. Although potential subjects were encouraged to participate in the study by informing them of the details of the study, privacy protection, and potential benefits, sampling bias may not be avoided entirely. Moreover, to ensure a certain level of representativeness, this study was mainly done in the three provinces in China (which is one of the provinces located in the east, central, and west of the Chinese economic areas, respectively), which still could not represent all the Chinese nurses and other health professionals. Additionally, these results could not be analogized to the general population. Another limitation of the study is that this is a cross-sectional descriptive study that limits causal inference between the risk factors and the occurrence of IP psychological violence. Finally, the measures of factors of a partner (e.g., alcohol consumption and personality traits) were proxy reported by respondents rather than from direct observations or reported by partners.

## 7. Conclusion

The present study identified the risk factors of IP psychological violence among Chinese nurses, namely, married but live apart, low contact frequency, high alcohol consumption frequency, past-year psychological and physical violence experience, low social support and low agreeableness, and emotional stability of partner. Structural modeling indicated that the personality traits of a partner might directly predict IP psychological violence and social support and indirectly predict psychological distress through IP psychological violence. In addition, social support may reduce IP psychological violence directly and further reduce psychological distress. The findings reported herein emphasize the possibility and importance of identification and intervention for reducing IP psychological violence based on personality traits and social support.

## Data availability statement

The raw data supporting the conclusions of this article will be made available by the authors, without undue reservation.

## Ethics statement

The studies involving human participants were reviewed and approved by First Affiliated Hospital of Guangdong Pharmaceutical University. The patients/participants provided their written informed consent to participate in this study.

## Author contributions

WH, FZ, YL, QY, XS, and YS made substantial contributions to the conception and design, or acquisition of data, or analysis and interpretation of data. WH, FZ, YL, and YS involved in drafting the manuscript or revising it critically for important intellectual content. WH, FZ, QY, XS, JH, YS, and YL given final approval of the version to be published, each author should have participated sufficiently in the work to take public responsibility for appropriate portions of the content, and agreed to be accountable for all aspects of the work in ensuring that questions related to the accuracy or integrity of any part of the work are appropriately investigated and resolved. All authors contributed to the article and approved the submitted version.
