# Supplementary material for: Association between intimate partner psychological violence and psychological distress among nurses: The role of personality traits and social support
Source: Front Psychol. 2023 Jan 12;13:1038428. doi: 10.3389/fpsyg.2022.1038428 (PMC9878691; doi:10.3389/fpsyg.2022.1038428)
Supplement: Supplementary file 1 [file Table_1.docx]

**Supplementary Table 1. Liner Regression on Selected Associated Factors for Psychological Violence among Nurses**

| Factors | **Unadjusted model** | **Fully adjusted model** | **Final adjusted model** |
| --- | --- | --- | --- |
|  | β（95%CI） | β（95%CI） | **β（95%CI）** |
| **Age (Continuous)** | 0.06 (0.03,0.09) | 0.05 (0.02,0.08) | 0.06 (0.03,0.08) |
| **Male (Ref: Female)** | 0.03 (-1.3,1.31) | -0.42 (-1.45,0.61) |  |
| **Have children (Ref: No)** | 0.79 (0.18,1.41) | 0.51 (-0.22,1.24) |  |
| **Married status (Ref: Marriage-cohabit)** | |  |  |
| Married, non-cohabit | 4.42 (3.13,5.72) | 1.19 (0.13,2.25) | 1.08 (0.04,2.12) |
| Unmarried, cohabit | 1.12 (-0.26,2.50) | 0.58 (-0.67,1.83) | 0.17 (-0.93,1.27) |
| Unmarried, non-cohabit | -0.48 (-1.31,0.34) | 0.21 (-0.69,1.11) | -0.23 (-0.95,0.49) |
| **Educational level (Ref: Technical secondary school)** | |  |  |
| Junior college | -0.75 (-2.81,1.31) | 0.02 (-1.54,1.58) |  |
| Undergraduate | -0.90 (-2.88,1.09) | -0.12 (-1.63,1.39) |  |
| Postgraduate | -0.92 (-3.40,1.55) | -0.29 (-2.17,1.60) |  |
| **Alcohol consumption (Ref: Lifetime abstainer)** | |  |  |
| Former drinker | 0.43 (-1.21,2.06) | -0.08 (-1.34,1.17) |  |
| <1 day/week | 0.48 (-0.10,1.06) | 0.06 (-0.40,0.52) |  |
| ≥1 day/week | 1.56 (-0.30,3.42) | 0.19 (-1.28,1.66) |  |
| **Partner alcohol consumption (Ref: Lifetime abstainer)** | |  |  |
| Former drinker | -0.69 (-2.12,0.75) | -0.47 (-1.59,0.65) | -0.32 (-1.41,0.77) |
| <1 day/week | 0.25 (-0.31,0.80) | -0.05 (-0.51,0.40) | -0.02 (-0.44,0.41) |
| ≥1 day/week | 1.78 (1.00,2.55) | 0.65 (0.00,1.29) | 0.69 (0.10,1.29) |
| **Contact frequency (Ref: Always)** |  |  |  |
| Often | 1.41 (0.89,1.93) | 0.69 (0.25,1.13) | 0.79 (0.36,1.22) |
| Occasionally | 2.26 (1.40,3.11) | 0.58 (-0.14,1.29) | 0.67 (-0.03,1.38) |
| Hardly ever | 5.78 (4.73,6.82) | 2.66 (1.75,3.57) | 2.88 (1.99,3.78) |
| **Past year violence experience (Ref: No)** | |  |  |
| Psychological violence | 4.51 (3.98,5.03) | 2.68 (2.16,3.20) | 2.74 (2.23,3.25) |
| Physical violence | 6.39 (5.04,7.74) | 2.18 (0.99,3.37) | 2.67 (1.56,3.78) |
| Sexual violence | 6.82 (4.53,9.12) | 1.85 (-0.05,3.75) |  |
| **Participant's Personalty traits (Continuous)** | |  |  |
| Extraversion | -0.13 (-0.24,-0.03) | 0.04 (-0.05,0.13) |  |
| Agreeableness | -0.34 (-0.47,-0.22) | 0.01 (-0.11,0.13) |  |
| Conscientiousness | -0.21 (-0.33,-0.09) | 0.05 (-0.07,0.16) |  |
| Emotional stability | -0.40 (-0.50,-0.29) | -0.03 (-0.14,0.08) |  |
| Openness | -0.09 (-0.22,0.04) | 0.07 (-0.04,0.19) |  |
| **Partner's Personalty traits (Continuous)** | |  |  |
| Extraversion | -0.20 (-0.30,-0.11) | -0.06 (-0.15,0.03) |  |
| Agreeableness | -0.70 (-0.79,-0.60) | -0.20 (-0.32,-0.07) | -0.21 (-0.32,-0.09) |
| Conscientiousness | -0.55 (-0.65,-0.45) | -0.08 (-0.18,0.02) |  |
| Emotional stability | -0.68 (-0.78,-0.58) | -0.16 (-0.28,-0.04) | -0.18 (-0.30,-0.07) |
| Openness | -0.35 (-0.46,-0.25) | 0.01 (-0.09,0.11) |  |
| **Social Support (Continuous)** |  |  |  |
| Objective support | -0.39 (-0.50,-0.27) | -0.16 (-0.27,-0.06) | -0.16 (-0.26,-0.06) |
| Subjective support | -0.25 (-0.31,-0.20) | -0.09 (-0.15,-0.04) | -0.09 (-0.14,-0.04) |
| Usage of support | -0.38 (-0.50,-0.26) | 0.00 (-0.11,0.12) |  |
| **Model Fit** |  |  |  |
| Adjusted R^2^ (%) |  | 44.59 | 44.72 |
| *F* |  | 20.93 | 43.58 |
| *p* |  | <0.001 | <0.001 |

Ref: reference variable in regression model.

Final adjusted model: eliminated the variables that were not statistically significant in the fully adjusted model.
