# Supplementary material for: Association between intimate partner psychological violence and psychological distress among nurses: The role of personality traits and social support
Source: Front Psychol. 2023 Jan 12;13:1038428. doi: 10.3389/fpsyg.2022.1038428 (PMC9878691; doi:10.3389/fpsyg.2022.1038428)
Supplement: Supplementary file 2 [file Table_2.docx]

**Supplementary Table 2. Liner Regression on Selected Associated Factors for Psychological Distress among Nurses**

| **Factors** | **Unadjusted model** | **Fully adjusted model** | **Final adjusted model** |
| --- | --- | --- | --- |
|  | β（95%CI） | β（95%CI） | **β（95%CI）** |
| **Age (Continuous)** | -0.09 (-0.17,-0.02) | -0.05 (-0.12,0.03) | -0.03 (-0.10,0.03) |
| **Male (Ref: Female)** | 2.84 (-0.06,5.74) | 1.41 (-1.14,3.96) |  |
| **Have children (Ref: No)** | -0.70 (-2.09,0.69) | 1.50 (-0.31,3.30) |  |
| **Married status (Ref: Marriage-cohabit)** | |  |  |
| Married, non-cohabit | 5.11 (2.14,8.09) | -0.32 (-2.95,2.31) |  |
| Unmarried, cohabit | 1.38 (-1.8,4.55) | -0.69 (-3.79,2.41) |  |
| Unmarried, non-cohabit | 1.70 (-0.2,3.59) | 0.99 (-1.25,3.23) |  |
| **Educational level (Ref: Technical secondary school)** | |  |  |
| Junior college | 1.34 (-3.30,5.98) | 2.51 (-1.36,6.37) |  |
| Undergraduate | 0.41 (-4.07,4.89) | 1.92 (-1.82,5.65) |  |
| Postgraduate | -0.65 (-6.23,4.93) | 1.59 (-3.08,6.25) |  |
| **Alcohol consumption (Ref: Lifetime obstainter)** | |  |  |
| Former drinker | 5.60 (1.95,9.25) | 4.27 (1.17,7.37) | 3.90 (0.86,6.94) |
| <1 day/week | 0.38 (-0.92,1.69) | 0.25 (-0.89,1.39) | 0.11 (-0.97,1.19) |
| ≥1 day/week | 5.90 (1.73,10.06) | 4.72 (1.09,8.34) | 4.91 (1.45,8.38) |
| **Partner alcohol consumption (Ref: Lifetime obstainter)** | |  |  |
| Former drinker | -1.65 (-4.91,1.62) | 0.02 (-2.74,2.79) |  |
| <1 day/week | -0.01 (-1.28,1.25) | -0.23 (-1.35,0.89) |  |
| ≥1 day/week | 2.09 (0.33,3.86) | -0.27 (-1.87,1.33) |  |
| **Contact frequency (Ref: Always)** |  |  |  |
| Often | 1.27 (0.02,2.52) | -0.31 (-1.41,0.79) |  |
| Occasionally | 4.65 (2.61,6.69) | 0.89 (-0.89,2.67) |  |
| Hardly ever | 4.29 (1.78,6.79) | -0.47 (-2.77,1.84) |  |
| **One year violence experience (Ref: No)** | |  |  |
| Psychological violence | 6.05 (4.75,7.35) | 1.29 (-0.08,2.65) | 1.23 (-0.09,2.55) |
| Physical violence | 6.50 (3.35,9.66) | 0.22 (-2.75,3.18) |  |
| Sexual violence | 7.50 (2.25,12.75) | 1.07 (-3.64,5.79) |  |
| **Psychological violence score(Continuous)** | 0.84 (0.70,0.98) | 0.38 (0.21,0.55) | 0.41 (0.25,0.56) |
| **Participant's Personalty traits (Continuous)** | |  |  |
| Extraversion | -0.80 (-1.02,-0.57) | -0.15 (-0.37,0.08) |  |
| Agreeableness | -1.3 (-1.58,-1.03) | -0.05 (-0.36,0.26) |  |
| Conscientiousness | -1.27 (-1.53,-1.00) | -0.23 (-0.52,0.05) |  |
| Emotional stability | -1.63 (-1.85,-1.41) | -0.76 (-1.04,-0.49) | -1.02 (-1.25,-0.79) |
| Openness | -1.01 (-1.3,-0.73) | -0.18 (-0.47,0.11) |  |
| **Partner's Personalty traits (Continuous)** | |  |  |
| Extraversion | -0.51 (-0.73,-0.28) | -0.11 (-0.33,0.11) |  |
| Agreeableness | -1.17 (-1.41,-0.93) | -0.09 (-0.40,0.21) |  |
| Conscientiousness | -1.19 (-1.41,-0.97) | -0.27 (-0.52,-0.01) | -0.36 (-0.58,-0.13) |
| Emotional stability | -1.18 (-1.41,-0.94) | -0.03 (-0.33,0.27) |  |
| Openness | -0.70 (-0.94,-0.46) | 0.14 (-0.12,0.40) |  |
| **Social Support (Continuous)** |  |  |  |
| Objective support | 0.13 (-1.27,-0.76) | -0.19 (-0.45,0.07) |  |
| Subjective support | 0.06 (-0.83,-0.6) | -0.24 (-0.38,-0.11) | -0.32 (-0.44,-0.20) |
| Usage of support | 0.14 (-1.58,-1.04) | -0.27 (-0.55,0.01) |  |
| **Model Fit** |  |  |  |
| Adjusted R^2^ (%) |  | 33.23 | 32.40 |
| *F* |  | 12.97 | 45.83 |
| *p* |  | <0.001 | <0.001 |

Ref: reference variable in regression model.

Final adjusted model: eliminated the variables that were not statistically significant in the fully adjusted model.
