# Supplementary material for: Association between intimate partner psychological violence and psychological distress among nurses: The role of personality traits and social support
Source: Front Psychol. 2023 Jan 12;13:1038428. doi: 10.3389/fpsyg.2022.1038428 (PMC9878691; doi:10.3389/fpsyg.2022.1038428)
Supplement: Supplementary file 3 [file Table_3.docx]

**Supplementary Table 3. Summary of standardized total effect and indirect effects of personality traits and social support on psychological distress.**

| **Pathway, X predict Y** | **Standardized Effects** |
| --- | --- |
| **Total Effect** |  |
| Partner Personality trait → Psychological distress | -0.186 |
| Social Support → Psychological distress | -0.353 |
| Participant Personality trait (ES)→ Psychological distress | -0.369 |
| **Indirect Effect (from Partner Personality trait)** |  |
| → Social Support →Psychological distress | -0.094 |
| → Psychological violence → Psychological distress | -0.081 |
| → Social Support →Psychological violence →Psychological distress | -0.011 |
| **Indirect Effect (from Social Support)** |  |
| →Psychological violence →Psychological distress | -0.036 |
| **Indirect Effect (from Participant Personality trait (ES))** |  |
| →Social Support →Psychological distress | -0.101 |
| →Social Support →Psychological violence →Psychological distress | -0.011 |
